# Measles Immune Suppression: Functional Impairment or Numbers Game?

**DOI:** 10.1371/journal.ppat.1004482

**Published:** 2014-12-18

**Authors:** Rory D. de Vries, Rik L. de Swart

**Affiliations:** Department of Viroscience, Erasmus MC, Rotterdam, The Netherlands; The Fox Chase Cancer Center, United States of America

## Introduction

Measles remains a significant cause of childhood morbidity and mortality. Hallmark of the disease is a generalized immune suppression that can last for several weeks to months after resolution of measles virus (MV) infection [1]–[3], resulting in increased susceptibility to opportunistic infections [4]–[7]. At the same time, measles is associated with immune activation and induces strong MV-specific immune responses that confer lifelong immunity [8]. This contradiction is known as the “measles paradox'. Although measles-associated immune suppression has been a subject of study since the beginning of the 20th century [9], the importance of possible underlying mechanisms remains disputed.

## The Immune System as “Viral Friend”

From the perspective of MV, cells of the immune system are both friend and foe. MV efficiently replicates in cells of the immune system, especially during the initial stages of the infection [10], [11]. However, the virus preferentially infects specific subsets of lymphocytes and dendritic cells (DCs). The relative susceptibility of these cells to MV infection is governed by their expression level of the cellular receptor CD150 [11]–[14]. Memory T-lymphocytes, which express CD150, are preferentially infected [13], [14]. In secondary and tertiary lymphoid tissues, the virus also replicates to high levels in follicular and marginal zone B-lymphocytes [10], [11], [13]. DCs can also be infected by MV [11], [15]–[17] and may serve as initial target cells [18], [19].

## The Immune System as “Viral Foe”

In the majority of cases MV infection is self-limiting and induces strong virus-specific cellular and humoral immune responses resulting in lifelong immunity [20]. Virus neutralizing antibodies are an important correlate of protection against MV infection, but cytotoxic T-lymphocytes are crucial for clearance of infected cells [21]–[23]. Resolution of MV infection is associated with increased lymphoproliferation [8], [24] and enlargement of lymph nodes [13]. Thus, the immune system efficiently restricts MV replication and clears MV-infected cells.

## Mechanisms of Measles Immune Suppression

Measles is associated with lymphopenia [25] and extensive depletion of lymphocytes from lymphoid tissues [13], [26], [27]. However, lymphocyte numbers return to normal within a week after clinical symptoms of measles have disappeared, while measles immune suppression extends for several weeks to months. Therefore, immune cell depletion was initially dismissed as a mechanism for measles immune suppression [3]. Alternative mechanisms have been proposed to explain measles-associated immune suppression, as summarized in Table 1. However, the relevance of these observations to enhanced susceptibility to opportunistic infections *in vivo* remains unclear.

**Table 1:** Reported mechanisms of measles immune suppression.

Functional Impairment	
*Mechanism*	*References*
Suppression of lymphocyte proliferation	[41]–[45]
Altered cytokine profiles	[42], [43], [46]–[50]
Lymphoproliferation defect due to MV-infected DC	[15], [17], [51]
Immune modulation mediated by viral proteins	[44], [52]–[56]
Modulation of cell membrane components	[57], [58]
Inhibition of hematopoiesis	[59], [60]

Depletion	
*Mechanism*	*References*
Lymphocyte infection & depletion	[11], [13], [14], [46], [61]–[64]
Bystander lymphocyte apoptosis	[65]–[67]
DC infection & depletion	[11], [16], [68], [69]
T-cell apoptosis after interaction with MV-infected DC	[16], [70]

## Is Suppressed Lymphoproliferation Important?

Reduced responsiveness of peripheral blood mononuclear cells to stimulation with mitogens *in vitro* has been considered an important mechanism underlying measles-associated immune suppression. Although the observations in these studies are not disputed, we find it difficult to reconcile this *in vitro* observation with the observed immune activation *in vivo*. Measles results in dramatic expansion of MV-specific lymphocytes followed by resolution of viremia and lymphopenia [8], [25], [28]. We recently demonstrated extensive lymphoproliferation in lymphoid tissues early after MV infection *in vivo* [13]. Thus, there is no evidence of suppressed lymphoproliferative responses, at least towards MV, *in vivo*. Rather, we believe that alterations in the composition of the peripheral lymphocyte populations before and after measles may explain these *in vitro* observations [13].

## Do Dendritic Dells Play a Crucial Role?

DC subsets have been shown susceptible to MV infection *in vitro* [15]–[17] and in nonhuman primates *in vivo* [11], [19]. Therefore, it is likely that infection, depletion, or functional modulation of DCs contributes to measles-associated immune suppression. Nevertheless, antigen presentation does not seem to be impaired *in vivo*, as strong MV-specific immune responses develop during the acute stage of the disease.

## Measles Damages the Respiratory Epithelium

Whereas MV targets CD150 to infect lymphoid and myeloid cells, the virus uses poliovirus receptor like 4 (also known as nectin-4) as an alternative cellular receptor to infect epithelial cells [29]–[31]. Whilst infection of epithelial cells contributes to viral transmission [32], MV also causes extensive epithelial damage in the respiratory tract [33], [34]. This epithelial injury may provide an opportunity for respiratory bacteria to adhere, replicate, and invade with increased efficiency [35].

## Attenuated, Mild, Moderate, or Severe Morbillivirus Infections

**Figure 1:**
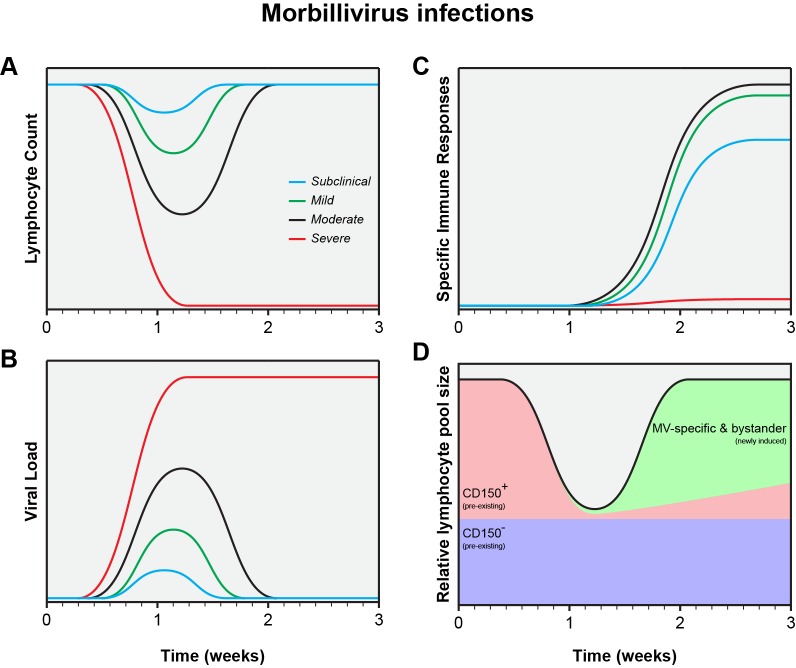
Schematic representation of the measles paradox. Different levels of lymphopenia (A), systemic virus loads (B), and virus-specific immune responses (C) after subclinical (blue), mild (green), moderate (black), or severe (red) morbillivirus infections. Panel D shows a model for immune suppression caused by moderate morbillivirus infection as shown in panels A, B, and C (adapted from [13]).

MV infections display a large variability in clinical severity, ranging from vaccination with attenuated viruses, via subclinical or mild infections, to severe disease. Closely related animal morbilliviruses may even overwhelm the immune system, resulting in functional paralysis and virtual absence of virus-specific immune responses [36]–[40]. This variation is also reflected in a wide range of levels of lymphopenia, viremia, and specific immune responses (Fig. 1A–C) [13]. Natural MV infection of the naive host will normally follow the pattern of either a mild or moderate infection as displayed in Fig. 1. Whereas mild measles results in limited depletion of pre-existing CD150^+^ memory lymphocytes, moderate measles is associated with infection and subsequent depletion of a large fraction of those lymphocytes (Fig. 1D). Whether this depletion is mediated by necrosis, apoptosis, pyroptosis, or cytotoxic T-cells remains to be determined, but the effect is always the same: to a varying degree, measles erases immunological memory.

## Future Directions: Studies in Naturally Infected Measles Patients

To improve our understanding of measles immune suppression, a transition from *in vitro* to *in vivo* studies is required. Two aspects are of crucial importance: viral tropism and depletion of immune cell subsets. We feel that it is important to characterize the phenotype of MV-infected cells during the prodromal phase of natural measles, with special emphasis on infection of DCs and memory lymphocytes. Furthermore, to address depletion of immune cell subsets, paired blood samples from children before and after measles will be required. Staining of immune cells specific for previously encountered pathogens, rather than functional assays, will allow us to distinguish between subset depletion and functional paralysis.

## Conclusions

Experimental MV infections in animal models have demonstrated that percentages of infected lymphocyte subsets are higher than previously thought, especially in secondary and tertiary lymphoid tissues [11], [13]. We believe that measles immune suppression mainly results from depletion of immune cell subsets, which is masked by the rapid proliferation of MV-specific and bystander lymphocytes (Fig. 1D). This model is fully compatible with the measles paradox. Clinical studies are required to test our hypothesis that measles immune suppression is mainly a numbers game.
